# Roles of mobile genetic elements and biosynthetic gene clusters in environmental adaptation of acidophilic archaeon *Ferroplasma* to extreme polluted environments

**DOI:** 10.3389/fmicb.2025.1654373

**Published:** 2025-07-31

**Authors:** Yiran Li, Liyuan Ma, Shanshan Huang, Shiqi Chen, Shadab Begum, Nazidi Ibrahim, Yili Liang, Xueduan Liu

**Affiliations:** ^1^School of Minerals Processing and Bioengineering, Central South University, Changsha, China; ^2^Key Laboratory of Biohydrometallurgy, Ministry of Education, Changsha, China; ^3^School of Environmental Studies, China University of Geosciences, Wuhan, China

**Keywords:** environmental adaptation, bioremediation, secondary metabolism, mobile genetic element, *Ferroplasma*

## Abstract

Acid mine drainage (AMD), characterized by high concentrations of heavy metals and strong acidity, presents a significant challenge in environmental remediation. The acidophilic archaeon *Ferroplasma* facilitates soluble electron shuttles secreting and iron precipitate formation to immobilize heavy metals and demonstrating significant remediation capabilities in microbial consortia. However, its environmental adaptation mechanisms in highly polluted environments during remediation remain unclear. Biosynthetic gene clusters (BGCs), which encode specialized metabolites with ecological roles, and mobile genetic elements (MGEs), known to mediate genomic function through gene disruption, rearrangement, and regulatory interference, represent crucial evolutionary means for environmental adaptation. In this study, *Ferroplasma acidiphilum* ZJ was screened from the traditional AMD of the Zijinshan copper mine, China. Then, it was sequenced, annotated and compared to three other sequenced *Ferroplasma* strains focusing on the distribution and function of genes concerning MGEs and BGCs. Genome-wide analysis indicated that MGEs, especially IS4 family insertion sequences (ISs) as well as genomic islands (GIs), were located close to functional regions, such as those related to heavy metal translocation, structural stability of cells, and the formation of archaeal ether-linked membranes. Further analysis showed *Ferroplasma* strains contained over 10 BGCs, with predicted functions spanning antibiotics, exopolysaccharide (EPS), and quorum sensing (QS). The *Ferroplasma* employed specialized MGEs and BGCs as key environmental adaptation mechanisms. This study provides a genetic framework for understanding the survival strategies of extremophiles in contaminated environments and explores the potential role of archaeal secondary metabolism (SM) in enhancing microbial processes for sustainable AMD bioremediation, by contributing to the detoxification and stabilization of heavy metals typically found in such environments.

## 1 Introduction

Microbial activities in disused mines can result in the dissolution of pyrite and other sulfides, generating significant quantities of acid mine drainage (AMD), a metal-laden acidic effluent that constitutes a substantial source of water contamination (Zhu et al., 2017). AMD significantly threatens public health in nearby communities through contamination of the food chain and drinking water, potentially causing heavy metal poisoning, skin diseases, and cancer (Wibowo et al., 2022). Among various remediation strategies, microbial-based bioremediation has gained prominence for its environmental friendliness, cost-effectiveness, and sustainability in addressing AMD pollution (Munyai et al., 2021). AMD remediation relies on the synergistic interactions of microbial communities. In these acidic environments, the archaeon *Ferroplasma* often forms competitive or symbiotic relationships with sulfur-oxidizing bacteria, collectively promoting ecosystem restoration (Ni et al., 2018). It is well documented that *Ferroplasma* are also capable of mobilizing metals from sulfide ores (e.g., pyrite, arsenopyrite, and copper sulfides), thereby affecting AMD formation (Johnson et al., 2012; Neculita et al., 2007; Ni et al., 2018). Additionally, *Ferroplasma* can mediate extracellular electron transfer, generating electric current and facilitating the sustainable removal of acid-generating inorganic sulfur compounds. This process underpins the use of Microbial Fuel Cell (MFC) biotechnology for AMD remediation (Ni et al., 2018). *Ferroplasma*, a wall-less archaea in the family *Ferroplasmaceae*, order Thermoplasmatales, was discovered in 2000 (Golyshina et al., 2000). It thrives in highly acidic conditions near pH 0, displaying a facultative heterotrophic/mixotrophic metabolism and iron oxidation ability (Dopson et al., 2004). This archaea is a dominant member of mixed-species biofilms, influencing iron and sulfur biogeochemical cycles in acidic ecosystems (Baker-Austin et al., 2010; Golyshina and Timmis, 2005). Consequently, elucidating the environmental adaptability of *Ferroplasma* is crucial for optimizing AMD bioremediation strategies, guiding the construction of more stable and efficient synthetic microbial consortia, and potentially uncovering metabolites with significant application value.

The environmental adaptation of microorganisms is a fundamental biological characteristic that enables their colonization and evolution in extreme habitats. Mobile Genetic Elements (MGEs) and secondary metabolites (SMs) that regulate biofilm formation are crucial adaptive strategies enabling acidophilic microorganisms to thrive in AMD (Aminov, 2011; Leal et al., 2020; Wang et al., 2010). MGEs include components such as Insertion Sequences (ISs), Genomic Islands (GIs), and CRISPR systems (Durrant et al., 2020). Through Horizontal Gene Transfer (HGT)-mediated genome reshaping, MGEs have become a driving force for microbial evolutionary adaptation (Guo et al., 2015; Huang et al., 2023). Preliminary genomic investigations of a *Ferroplasma* isolate have revealed characteristic MGE features, including metal resistance operons preferentially localized within GI regions and conserved toxin-antitoxin modules (Bargiela et al., 2023). It is essential to systematically compare the distribution of ISs and GIs across different *Ferroplasma* strains and identify the specifically enriched functional genes. The quorum sensing (QS)-dependent biofilm regulatory mechanism offers a distinctive adaptation strategy for *Ferroplasma* in extremely acidic environments (Huang et al., 2022). While QS signal molecules have been extensively studied in the biofilm development of other extremophiles, the *Ferroplasma* genome lacks typical QS-related molecular features (Luo et al., 2024). Instead, its biofilm formation may rely on a novel regulatory network mediated by SMs. Identifying the biosynthetic gene clusters (BGCs) encoding these SMs and analyzing their roles in biofilm matrix assembly, cell attachment, heavy metal adsorption, and interspecies interactions could elucidate the unique QS regulatory strategies employed by this acidophilic archaeon.

Inadequate genomic data impedes exploration of *Ferroplasma*’s metabolic diversity and adaptive strategies, including its metabolic flexibility in natural environments and potential use of extracellular electron transfer in MFC technology. Metagenomic studies highlight genomic diversity in coexisting *Ferroplasma* populations due to evolutionary processes (Golyshina, 2011; Tokuda and Shintani, 2024). Studies primarily focus on model strains *F. acidiphilum* Y and *F. acidarmanus* fer1, revealing cultivation challenges in this archaeal genus (Dopson et al., 2004; Eppley et al., 2007; Golyshina et al., 2017; Yelton et al., 2013). Further investigation is needed to understand the roles of horizontal gene transfer and genomic plasticity in niche differentiation and functional division among strains in mixed biofilms, utilizing integrated multi-omics approaches like single-cell genomics.

The acidophilic archaeon *Ferroplasma acidiphilum* ZJ, isolated from the Zijinshan copper mine (China), demonstrates exceptional acid tolerance with growth capabilities in extreme acidic conditions (pH 0.5–1.5). We conducted a comprehensive pangenomic analysis of available *Ferroplasma* genomes. Through integrated average nucleotide identity (ANI) and pangenome profiling, we resolved taxonomic classifications while identifying MGEs, including ISs and GIs, as key mediators of adaptive evolution. Notably, we examined BGCs to assess the potential involvement of horizontal gene transfer (HGT) in environmental adaptation.

## 2 Materials and methods

### 2.1 Strain isolation and cultivation

In this study, strain ZJ was isolated from AMD samples collected on August 15, 2022, from the Zijinshan copper mine (25°10′41”N-25°11′44”N, 116°24′00″E-116°25′22″E) in Fujian Province, China. The isolation process involved inoculating 10.0 mL aliquots of liquid samples into modified 9K medium (pH 1.6) supplemented with 22.4 g/L FeSO_4_·7H_2_O and 0.02% (w/v) yeast extract. Cultivation was conducted at 40°C under aerobic conditions using a rotary shaker at 170 rpm. The base 9K medium composition consisted of (per liter): (NH_4_)_2_SO_4_ (3.00), K_2_HPO_4_ (0.50), KCl (0.10), Ca (NO_3_)_2_ (0.01), and MgSO_4_·7H_2_O (0.50). Following primary enrichment, strain ZJ was purified through serial 10-fold dilutions in sterile 9K and subsequently subjected to genome sequencing.

### 2.2 DNA extraction, sequencing, annotation, and phylogenomic analyses

The genomic DNA of *F. acidiphilum* ZJ was isolated from late-exponential phase cultures using centrifugation (12,000 × g for 10 min at 4°C) followed by purification with a QIAamp DNA Mini Kit (Qiagen, Germany) according to manufacturer protocols. DNA quantification was performed fluorometrically using a TBS-380 instrument (Turner BioSystems, United States), with high-purity samples (OD260/280 = 1.8–2.0, >10 μg) subsequently subjected to hybrid sequencing through third-generation PacBio RS and second-generation Illumina HiSeq platforms (Shanghai Majorbio Bio-pharm Technology Co., China) (Yang et al., 2024).

Raw sequencing data from both platforms were subjected to quality filtering to generate clean reads. *De novo* genome assembly was performed through a hierarchical approach combining the HGAP pipeline and Canu assembler with default parameters (Chin et al., 2013; Koren et al., 2017), followed by manual verification of terminal cycling steps to ensure chromosomal completeness. Assembly refinement using Illumina reads was conducted with Pilon (Koren et al., 2017; Walker et al., 2014), with all analyses executed on the Majorbio Cloud Platform. Gene prediction employed Glimmer v3.02 (Delcher et al., 2007), retaining open reading frames (ORFs) > 300 base pairs (bp) for functional annotation through BLASTP searches against NCBI-nr, Swiss-Prot, KEGG, and COG databases. Comprehensive genome annotation was achieved through RAST^1^ (Overbeek et al., 2014), incorporating rRNA identification via Barrnap v0.9 and tRNA detection using tRNAscan-SE v1.23 (Chan et al., 2021). KEGG database analysis enabled metabolic pathway reconstruction through systematic mapping of KO identifiers (K numbers) to organism-specific GENES identifiers (Kanehisa et al., 2023), supported by whole-genome comparisons against NCBI nucleotide collections (Federhen, 2012).

Phylogenetic reconstruction utilized two complementary approaches: an alignment-free composition vector method implemented in CVTree4 (Zuo, 2021) and maximum likelihood analysis conducted in MEGA v11 (Tamura et al., 2021). Final tree visualization and annotation were performed using the web-based Chiplot platform (Xie et al., 2023), ensuring robust phylogenetic resolution through dual methodological validation.

### 2.3 Comparative genomics analysis

For comparative genomic analysis within the *Ferroplasma* genus, four complete genomes were selected based on phylogenetic relationships: *F. acidiphilum* ZJ, *F. acidarmanus* fer1, *F. acidiphilum* Y, and *F. acidiphilum* DSM 28986. Orthologous Average Nucleotide Identity (OrthoANI) values between these strains were calculated through whole-genome comparisons using two independent online platforms, JSpeciesWS and Ezbiocloud. The resulting ANI matrices were visualized as heatmaps through the OAT: OrthoANI toolkit (v0.93.1) (Lee et al., 2016). Genome annotation was performed using Prodigal (v2.6.3) (Hyatt et al., 2010) for consistent gene prediction across all strains.

Pangenome analysis was conducted using the Bacterial Pan Genome Analysis tool (BPGA v1.3) with the integrated USEARCH program (v11.0.667) (Chaudhari et al., 2016). A sequence identity threshold of 50% was applied for clustering homologous genes. The BPGA pipeline generated mathematical models describing core and pangenome dynamics through the power-law function *y* = ax^b^, where parameter b indicates genome openness. Values of 0 < *b* < 1 characterize an open pangenome, suggesting continuous acquisition of novel genes when new genomes are added. Conversely, *b* ≥ 1 indicates a closed pangenome, where additional genomes do not substantially increase genetic diversity, reflecting limited environmental adaptability through gene exchange.

### 2.4 Identification of MGEs

The identification and classification of ISs within the *F. acidiphilum* ZJ genome were conducted using the ISfinder platform, with subsequent validation through online BLAST analysis employing a stringent *E*-value threshold of 1e^−10^ (Siguier et al., 2006). Genomic coordinates, family classification, and copy numbers of all IS elements were systematically retrieved from the BLAST outputs. A comprehensive physical gene map was constructed by mapping functional regions adjacent to the IS elements. GI prediction was performed through IslandViewer 4 (Bertelli et al., 2017) with three detection algorithms: IslandPath-DIMOB, IslandPick, and SIGIHMM. Proteins encoded within GIs identified by at least one prediction method were subsequently cataloged for functional annotation.

### 2.5 Identification of BGCs

The BGCs in four *Ferroplasma* strains were identified using the web-based platform antiSMASH 7.0 (Blin et al., 2023), the predominant bioinformatics resource for BGC prediction and characterization in archaeal genomes. This tool integrates detection rules for 81 distinct BGC types (Blin et al., 2021) and was configured with the following parameters: relaxed detection stringency, activation of all optional features, and inclusion of computationally intensive analytical modules. Input data comprised genomic sequences in FASTA format and their corresponding structural annotations in GFF3 format. Following computational analysis, the detected BGCs were classified into seven major categories: non-ribosomal peptide synthetases (NRPS, including NRPS-like variants), polyketide synthases (PKS), hybrid Fix clusters, ribosomally synthesized and post-translationally modified peptides (RIPP), terpenes, alkaloids, and an “others” group. The latter category encompassed rare biosynthetic pathways such as phosphonate, NI-siderophore, cyclic dipeptide synthase, and phosphonate-like systems (Li et al., 2021). For comparative analysis, the functional diversity of BGCs across strains was visualized using FigDraw to generate a structured representation of their metabolic potential.

## 3 Results and discussion

### 3.1 Genomic features of strain ZJ

**Figure 1:**
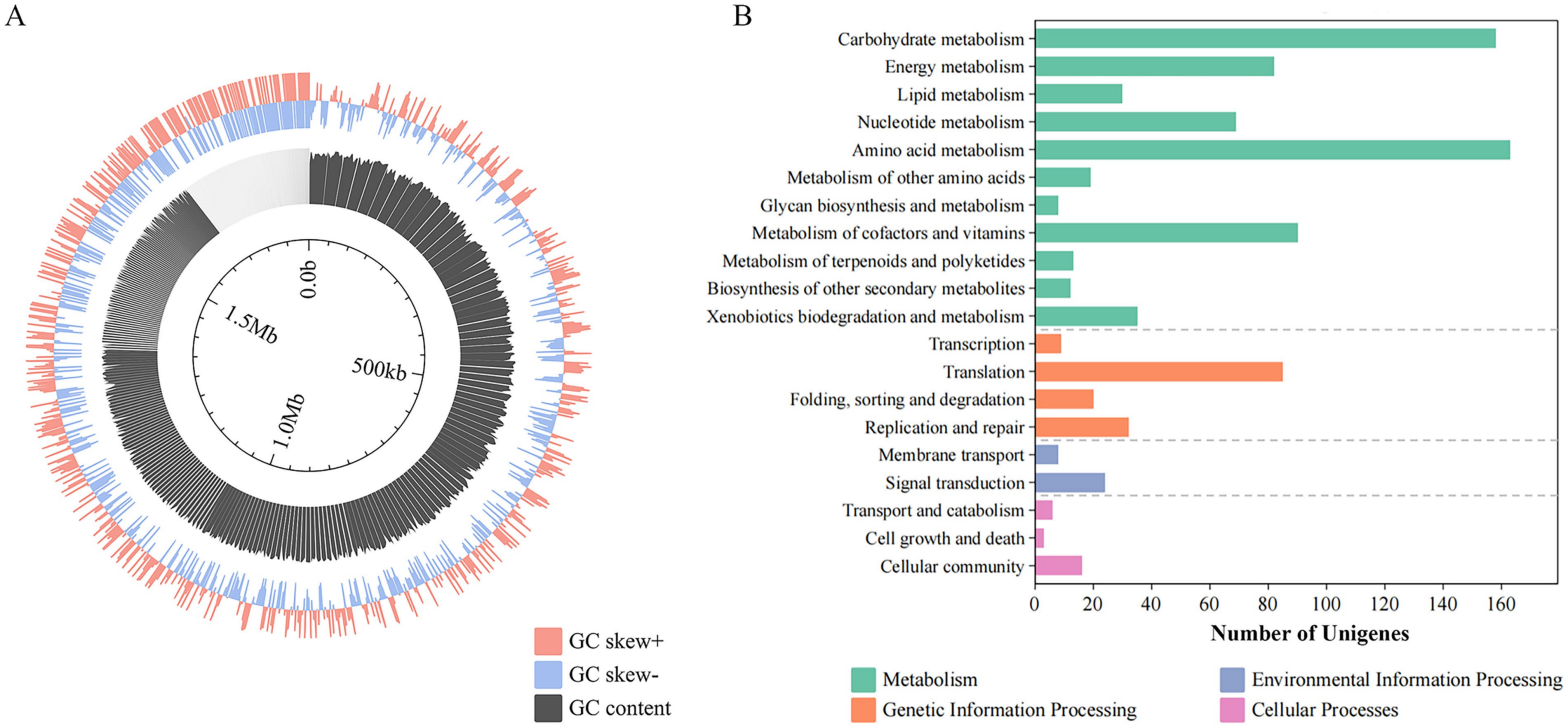
Genomic features of strain ZJ. **(A)** Genome circos of strain ZJ. **(B)** KEGG functional classification of strain ZJ, including metabolism, genetic information processing, environmental information processing, and cellular processes.

The genome comprises a single circular chromosome spanning 1,804,513 bp with a GC content of 36.95%, assembled into 413 contigs (Figure 1A). Key genomic features include 43 tRNA genes, 2,084 protein-coding sequences (CDSs), and five GIs, while CRISPR arrays were not detected through computational prediction. Functional annotation through KEGG classification revealed 1,117 genes distributed across four primary categories: metabolism, genetic information processing, environmental information processing, and cellular processes (Figure 1B). Within metabolic pathways, amino acid metabolism constituted the most gene-enriched subsystem. The genetic information processing category showed translational machinery components as the most abundant functional group, followed by homologous recombination and DNA repair systems, which might enhance the DNA maintenance capacity. Notably, cellular process annotations revealed a substantial proportion of genes associated with microbial interactions, suggesting potential ecological competence through intercellular communication mechanisms in strain ZJ’s native environment.

Quorum sensing signaling molecules (QSSM), functioning as bioactive natural products, critically regulate biofilm formation and microbial community interactions. Genomic characterization of strain ZJ demonstrated that the majority of its QS-associated genes encode two key enzymatic components: PhnA and PhnB responsible for *Pseudomonas* quinolone signal (PQS) biosynthesis, along with RpfB implicated in the diffusible signal factor (DSF) system. Of particular significance, both PQS and DSF-mediated regulatory systems have been established as pivotal signaling mechanisms governing microbial behavior in acidophilic environments, as evidenced by previous research (Huang et al., 2022).

### 3.2 Genome features and comparative genomics of *Ferroplasma*

#### 3.2.1 Genome statistics

Through 16S rRNA research, it was found that ZJ belongs to the genus *Ferroplasma* (Supplementary Figure S1). To investigate environmental adaptation mechanisms within *Ferroplasma*, we conducted a comparative genomic analysis of all isolated whole-genome sequenced strains. The examined strains exhibited conserved genomic characteristics, as evidenced by their comparable GC content and genome architecture (Table 1). Notably, all four strains maintained narrow GC content ranges between 36.50 and 36.95%, with genome sizes averaging 1.85 Mbp. The predicted protein-coding gene counts demonstrated moderate variation across strains, ranging from 1,842 to 2,084 genes. These conserved genomic parameters suggest evolutionary stability in fundamental genomic organization among *Ferroplasma* species, while the observed gene content variations may reflect niche-specific adaptations.

**Table 1:** Overview of *Ferroplasma* genomes used in this study.

Strain	Size (Mbp)	Level	GC%	GenBank assembly
*Ferroplasma acidiphilum* ZJ	1.80	Contig	36.95	GCA_050311795.1
*Ferroplasma acidarmanus* fer1	1.94	Complete	36.50	GCA_000152265.2
*Ferroplasma acidiphilum* Y	1.83	Complete	36.50	GCA_002078355.1
*Ferroplasma acidiphilum* DSM 28986	1.81	Contig	36.90	GCA_013133875.1

**Figure 2:**
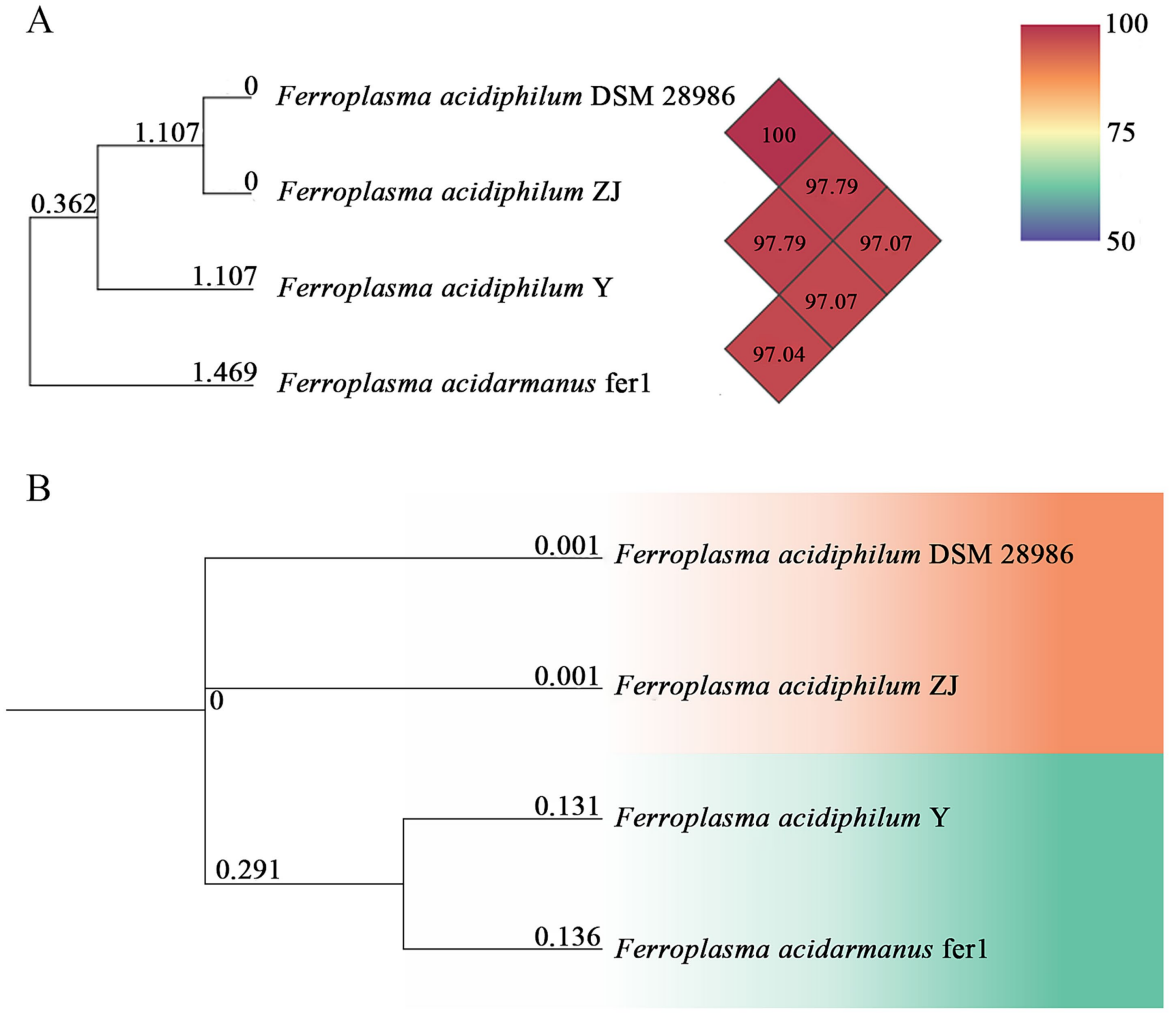
Heatmap for OrthoANI values between pairs of *Ferroplasma* strains using OAT software **(A)**. Whole-genome-based phylogenetic tree of *Ferroplasma* strains with neighboring standard species **(B)**.

The ANI serves as a robust genomic metric for evaluating phylogenetic relatedness between microbial strains, with established thresholds defining species boundaries (de Albuquerque and Haag, 2023). Conventionally, ANI values exceeding 95% are considered indicative of strains belonging to the same recognized microbial species (Luis et al., 2023). Our analysis revealed observed ANI values between the newly isolated strains ZJ and *F. acidiphilum* exceeding this benchmark (Figure 2A), demonstrating their close phylogenetic affiliation. Notably, the calculated ANI between *F. acidiphilum* DSM 28986 and strain ZJ reached 100% genomic identity, strongly supporting their classification as conspecific strains according to current taxonomic standards (Viver et al., 2024). This genomic identity threshold corroborates their taxonomic assignment within the same species despite potential phenotypic variations.

Based on comprehensive phylogenetic analysis of complete genome sequences performed in this study, we propose that strain ZJ should be taxonomically classified within *F. acidiphilum* species, as evidenced by its consistent clustering with reference strains in the phylogenetic reconstruction (Figure 2B) (Hu et al., 2024).

#### 3.2.2 Pangenome analysis

**Figure 3:**
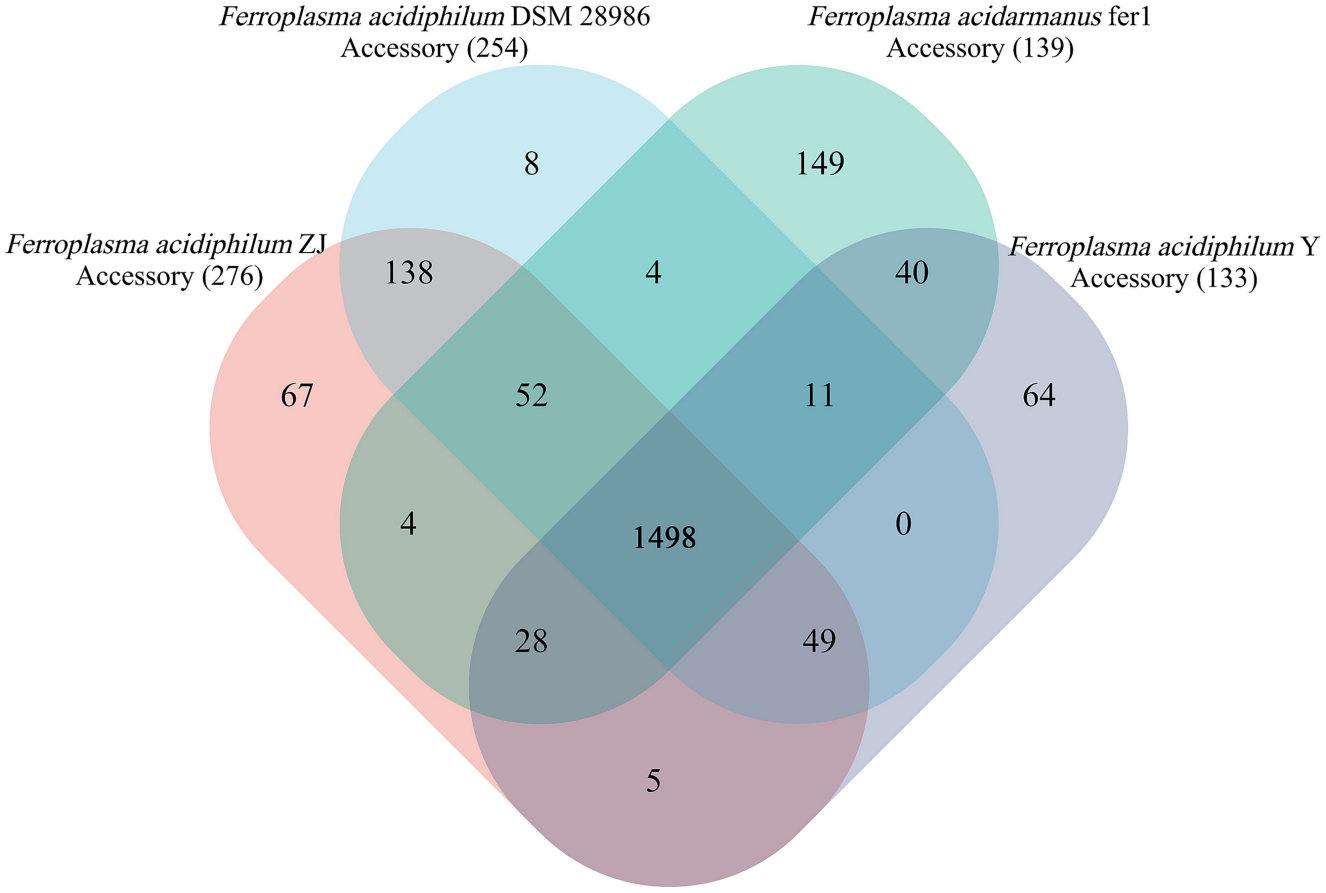
Pangenome analysis of *Ferroplasma* strains. The numbers in the center of Venn diagram are the numbers of core genes. The numbers on the flower petals indicate the number of unique genes. Other areas are locations of accessory genes.

As illustrated in Figure 3, pangenome analysis conducted on the four *Ferroplasma* strains delineated three distinct genomic components: the core genome, accessory genome, and unique genome (Horesh et al., 2021). Subsequent COG functional annotation revealed characteristic distribution patterns of COG categories across these genomic compartments. While the current analysis suggests an open pan-genome state, this configuration may transition to a closed state with expanded sampling. Notably, *Ferroplasma* exhibits evolutionary flexibility through HGT mechanisms, enabling interspecies acquisition of MGEs that contribute to its adaptive genomic repertoire (Vernikos et al., 2015). This metabolic plasticity is particularly significant given the organism’s extreme acidophilic habitat requirements.

**Figure 4:**
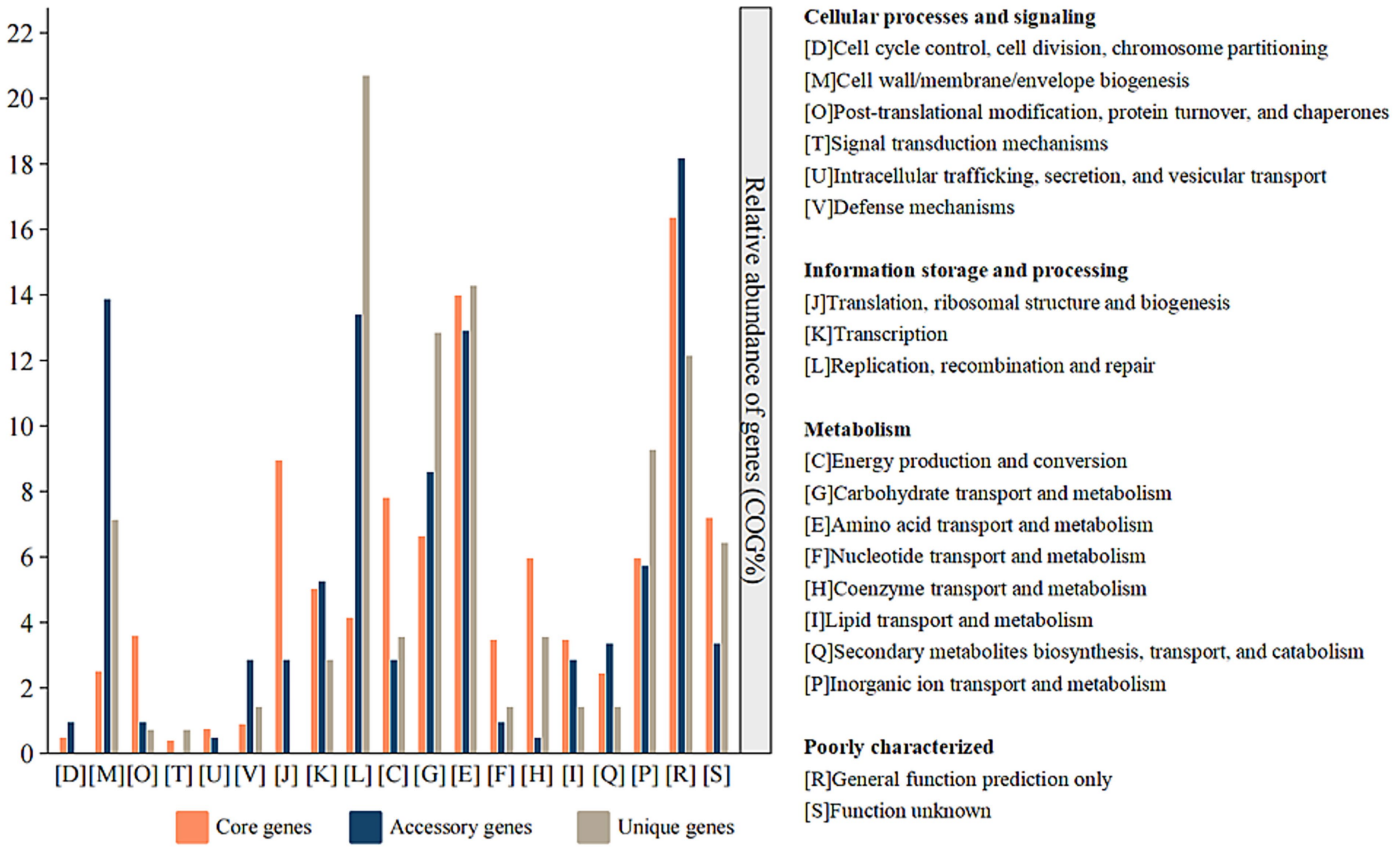
COG functional classification of core, accessory and unique genes in *Ferroplasma* strains.

The core genome of *Ferroplasma* comprises 1,498 CDSs, with *F. acidiphilum* ZJ harbored 276 accessory genes among studied species. These accessory genomic elements, known to regulate genetic traits associated with environmental adaptability, stress resistance, and virulence (Medini et al., 2005), contribute significantly to intraspecies diversity. Compared with the genomes from the *Ferroplasma*, *F. acidiphilum* ZJ harbored the largest accessory genes (276 genes) apart from other genomes, implying a relatively high degree of genomic diversity. Functional annotation of accessory genes (Figure 4) reveals three predominant categories: [M] cell wall/membrane/envelope biogenesis, [V] defense mechanisms, and [Q] secondary metabolites biosynthesis, transport, and catabolism. This distribution may suggest evolutionary specialization through structural adaptations (particularly in cellular envelope components) and sophisticated defense systems to counter extreme pH stress. Notably, SMs appear to play pivotal roles in *Ferroplasma*’s unique environmental adaptation strategies. The observed genomic diversity and functional specialization collectively provide a competitive advantage for niche colonization in harsh ecosystems, demonstrating a robust evolutionary framework for extremophile survival.

### 3.3 MGE identification and adaptive evolution in *Ferroplasma*

#### 3.3.1 ISs in *Ferroplasma*

A comprehensive analysis of IS elements across *Ferroplasma* genomes revealed nine distinct IS families comprising 195 total copies, as detailed in Supplementary Table S1. Comparative genomic profiling demonstrated significant interspecies variation, with reference strains *F. acidarmanus* fer1 (76 copies) and *F. acidiphilum* Y (70 copies) harboring substantially higher IS loads compared to *F. acidiphilum* DSM 28986 (27 copies) and *F. acidiphilum* ZJ (22 copies). Six predominant families (IS256, IS200/IS6, IS5, IS4, ISH3, and IS481) collectively accounted for >85% of IS elements across the four genomes. Core IS families (IS4, ISH3, IS200/IS6, IS256, IS481, IS5, and IS110) were conserved in all strains, while strain-specific distributions emerged for IS1634 (exclusive to *F. acidiphilum* Y and *F. acidarmanus* fer1) and ISNCY (unique to *F. acidarmanus* fer1). Notably, IS4 demonstrated universal distribution and particular functional relevance, potentially serving as an evolutionary toolkit through HGT to enhance microbial adaptation in extreme environments, as evidenced by its prevalence in extremophiles and pathogenic bacteria (Lysnyansky et al., 2009; Touchon and Rocha, 2007). Mechanistic studies suggest IS4 may modulate salinity tolerance modulation and genomic plasticity in response to environmental stressors (Bonnefoy et al., 2018).

**Figure 5:**
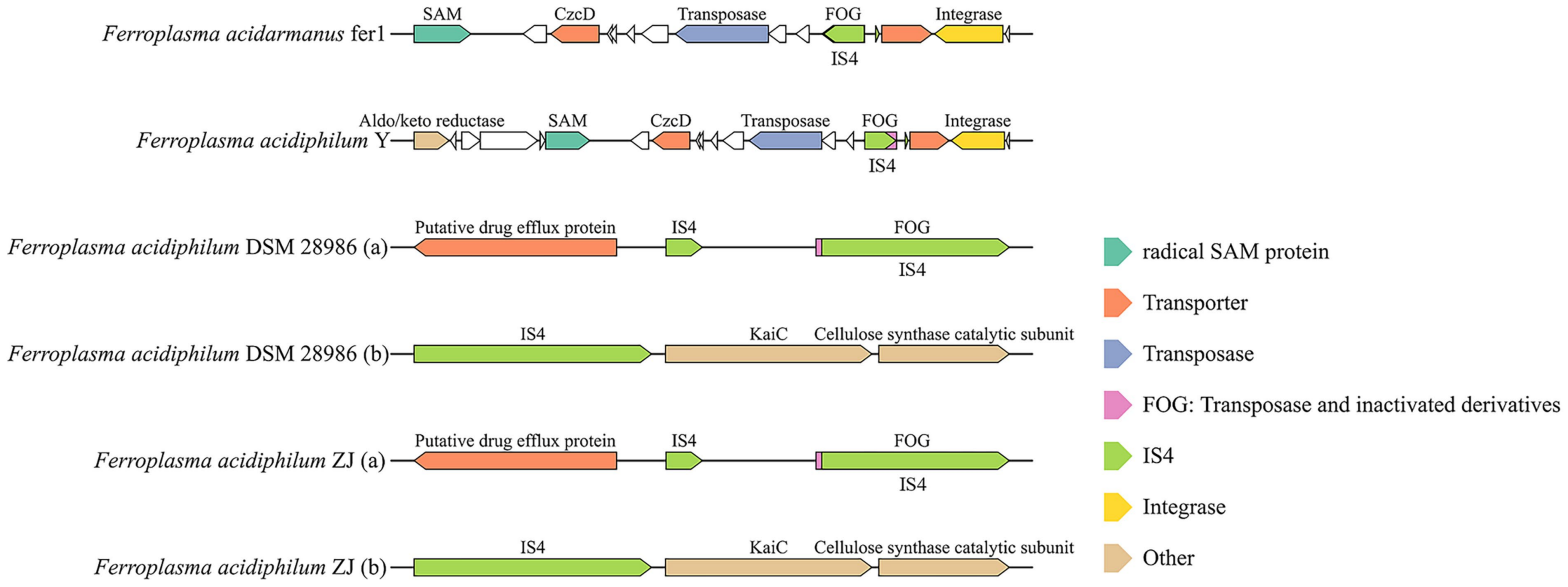
Putative laterally transferred regions containing IS4 family in *Ferroplasma* strains.

Previous studies proved that the insertion and deletion of IS4 in specific sequences affects the ability of extreme acidophilies in the AMD environment, to utilize iron (Bonnefoy et al., 2018). Functional characterization of IS4 through adjacent gene analysis revealed distinct genomic insertion patterns (Figure 5). Unlike other IS families, IS4 frequently co-occurred with FOG (Functional Orthologous Group) transposase derivatives, suggesting potential archaeal-specific transposition mechanisms. Comparative mapping identified preferential IS4 insertion near critical metabolic loci, including: (1) heavy metal transporter genes (e.g., CzcD transporters for cobalt/zinc/cadmium detoxification) (Hassan et al., 2013); (2) membrane biosynthesis components (Radical SAM proteins and aldo/keto reductase for QS molecule synthesis in *F. acidiphilum* Y and *F. acidarmanus* fer1); and (3) structural elements supporting biofilm maintenance (cellulose synthase catalytic subunits in *F. acidiphilum* ZJ and DSM 28986 genomes) (Römling and Galperin, 2015). These strategic insertion patterns indicated that IS4-mediated genomic restructuring enhances environmental adaptability through several mechanisms: improving heavy metal transport, optimizing conversion rates, reinforcing cell membrane integrity, and augmenting specialized metabolic functions. The distribution pattern of IS4 and its association with gene neighborhoods offer mechanistic insights into how MGEs influence the functional genomic evolution of extreme acidophilic archaea and enhance their environmental remediation capabilities.

#### 3.3.2 GIs in *Ferroplasma*

Five GIs ranging from 11.5 to 51.0 kb were identified in the *F. acidiphilum* ZJ genome (Supplementary Figure S2), harboring 159 predicted genes. Comparative analysis revealed interspecies variability in GI content: *F. acidiphilum* DSM 28986 contained the highest gene count (210 genes) distributed across 8 GIs, while *F. acidarmanus* fer1 exhibited the most extensive GI architecture with 11 GIs encompassing 176 genes. *F. acidiphilum* Y displayed intermediate characteristics, with 173 genes organized into 7 GIs (Supplementary Table S2). Subsequent functional annotation highlighted a predominance of MGE-associated genes, including 18 coding for transposases, integrases, and recombinases. This finding aligns with the established role of GIs in facilitating HGT through transformation, conjugation, and transduction mechanisms (Juhas et al., 2009). Additionally, genes encoding transferases, hydrolases, and transporter proteins were identified. Notably, a CRISPR-associated Cas1 protein was detected within *F. acidiphilum* ZJ’s GI, suggesting potential CRISPR-mediated defense against MGEs or phage infection. However, no complete CRISPR arrays were identified in the genome. The analysis further predicted five restriction-modification system subunits within GIs, implicating these regions in restriction-based defense systems. Of particular ecological relevance, heavy metal resistance genes were systematically identified across all *Ferroplasma* strains. This included the conserved cobalt/zinc/cadmium resistance protein CzcD, a cation-transporting transmembrane protein previously characterized in acidophilic archaea (Deppenmeier et al., 2002). Heavy metal-binding proteins encoded in the *Ferroplasma* archaea genome are crucial for mitigating heavy metal toxicity. These proteins safeguard cellular structure and function by actively exporting heavy metal ions, preventing their accumulation to toxic levels. Toxin-Antitoxin systems are widely present in archaea and typically consist of two genes: one encodes a toxin and the other encodes an antitoxin. In addition to this, the prevalence of efflux pumps, transporters, and heavy metal resistance factors in GIs underscores their role in adapting to extreme environments and suggests potential applications in environmental remediation.

##### 3.3.2.1 Radical SAM protein and calditol control the formation of the archaeal membrane

The Radical SAM protein encoded by the conserved CDS plays an essential role in calditol biosynthesis, with calditol-linked glycerol dialkyl glycerol tetraethers constituting up to 90% of polar lipids in *Sulfolobus acidocaldarius* (Zeng et al., 2018). This radical SAM-dependent modification of archaeal membrane lipids imparts protective advantages against environmental pH fluctuations. Specifically, calditol-containing lipids demonstrate significant acid-stress mitigation capabilities in *S. acidocaldarius*, as evidenced by their membrane-stabilizing function under extreme acidic conditions (Zeng et al., 2018). Genomic analysis reveals the radical SAM protein-encoding gene resides within GIs of all *F. acidiphilum* strains (ZJ, DSM 28986, and Y) (She et al., 2001). Biochemical characterization confirms the enzyme’s catalytic activity through iron–sulfur cluster coordination and metal ion binding, demonstrating dual functionality in both heme biosynthesis and membrane lipid modification (Li et al., 2024; Lobo et al., 2014). These findings establish that radical SAM-mediated calditol incorporation into GDGTs provides critical structural reinforcement and functional optimization of archaeal membranes. Therefore, this might be a likely necessary adaptation mechanism for extremophile survival in highly acidic environments. The conserved presence of this system across acidophilic archaea underscores its evolutionary importance in maintaining membrane integrity under proton stress conditions.

##### 3.3.2.2 Aldo/keto reductase and QS

The gene encoding “Aldo/keto reductase” has been identified in multiple strains of the iron-reducing hyperthermophilic archaeon *Saccharolobus solfataricus* and universally detected within the GIs of all *F. acidiphilum* strains (Penning, 2015). Notably, a functionally characterized aldo-keto reductase (VCA0859) in *Vibrio cholerae* has been demonstrated to catalyze the biosynthesis of CAI-1 (Cholera Autoinducer-1), a QSSM employed by bacteria for intraspecific and interspecific communication (Jahan, 2011). Experimental evidence demonstrates its regulatory role in modulating biofilm formation pathways in *V. cholerae*, with additional capacity to influence the QS regulatory network (Jahan, 2011). Collectively, these findings support the hypothesis that aldo/keto reductase family members may serve critical functions in both microbial communication systems and environmental adaptation mechanisms, potentially through their involvement in secondary metabolite synthesis and stress response pathways.

### 3.4 BGC identification and environmental adaptation of *Ferroplasma*

**Figure 6:**
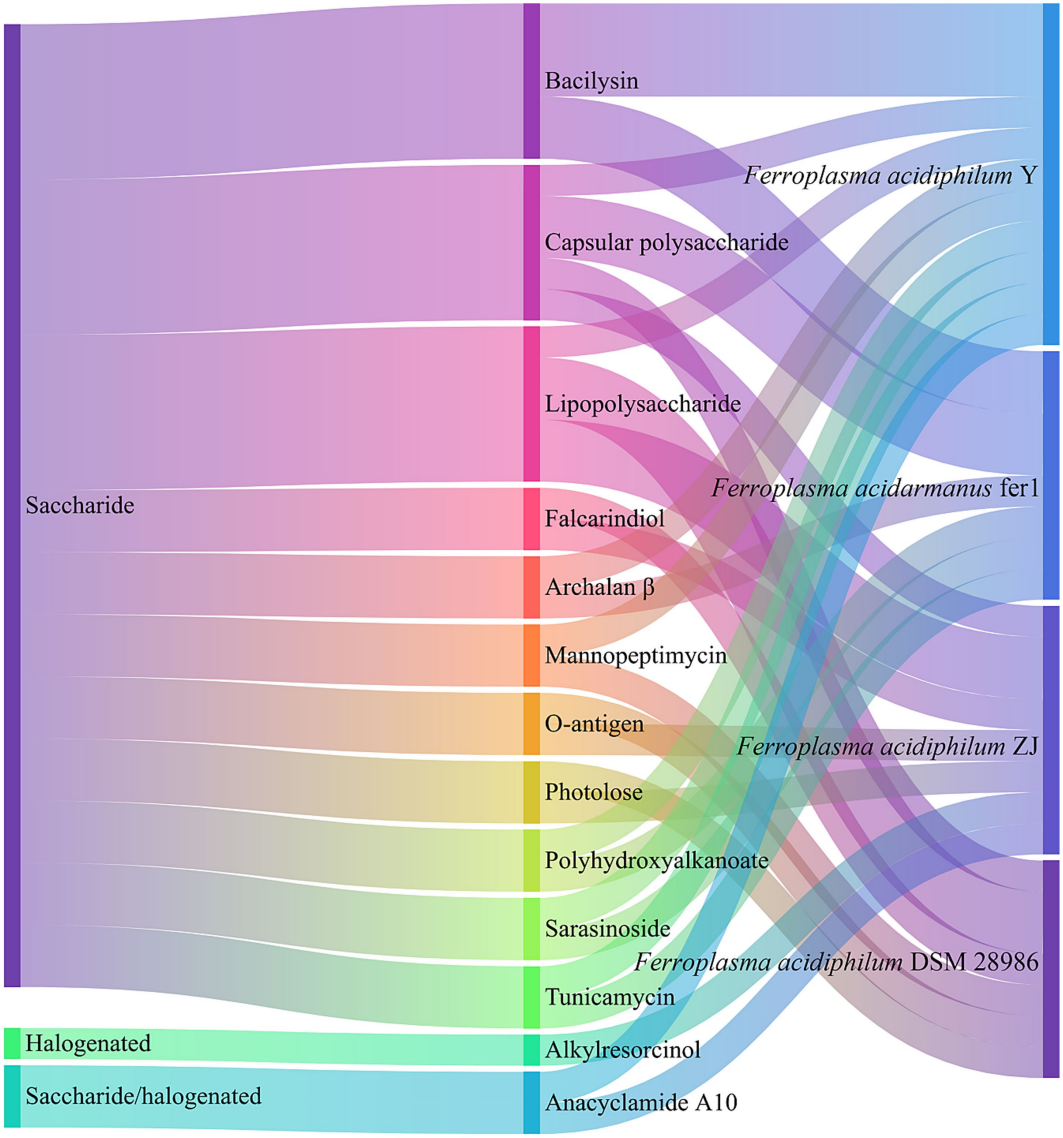
Classification of BGCs in *Ferroplasma*.

As detailed in Figure 6, BGCs in archaea encode diverse saccharide compounds including capsular polysaccharides, lipopolysaccharides, and O-antigens, along with structurally varied halogenated. Notably, these archaeal BGCs demonstrate significant potential for producing antimicrobial agents, with computational predictions indicating numerous clusters associated with antibacterial peptides and antibiotics (Perry et al., 2022). A prominent example is bacilysin, a structurally simple yet potent peptide antibiotic composed of L-alanine and the non-proteinogenic amino acid L-anticapsin (Steinborn et al., 2005). This compound exhibits broad-spectrum antimicrobial activity through targeted inhibition of glucosamine-6-phosphate synthase, a critical enzyme in bacterial peptidoglycan and fungal mannoprotein biosynthesis (Chmara and Borowski, 1986). The BGC of *Ferroplasma* further encompasses other bioactive clusters encoding tubercidin, tunicamycin, mannopeptimycin, and archalan *β*, and the latter representing archaea-specific bacteriostatic compounds.

Functional analysis reveals additional BGCs potentially involved in synthesizing membrane-modulating branched-chain fatty acids (influencing membrane fluidity) (Mindrebo et al., 2020), stress-protective polyhydroxyalkanoates (Li et al., 2016), and virulence-associated pseudaminic acid glycoconjugates, the latter particularly significant in Gram-negative pathogens like *Acinetobacter baumannii* for surface structure formation (Zamora et al., 2017). In the case of *F. acidiphilum* ZJ, genomic characterization identified 10 chromosomally encoded secondary metabolite clusters, including those producing quorum-sensing inhibitor (QSI) such as falcarindiol. However, a substantial proportion of *Ferroplasma* BGCs remain functionally uncharacterized, with clusters tentatively linked to photolose, anacyclamide A10, and alternative PHA synthesis pathways currently pending further investigation.

This systematic annotation highlights both the chemical diversity encoded in archaeal genomes and the significant knowledge gaps requiring experimental validation, particularly regarding novel BGC functions and their ecological restoration roles in extremophilic organisms.

#### 3.4.1 Exopolysaccharide (EPS), adsorption of heavy metals

The prediction of secondary metabolite synthesis gene clusters in three *F. acidiphilum* strains (ZJ, DSM 28986, and Y) revealed their ability to produce two types of exopolysaccharides: lipopolysaccharide and capsular polysaccharide. Different from other strains, *F. acidarmanus* fer1 can only synthesize lipopolysaccharide and lacks the ability to synthesize capsular polysaccharide. The absence of a cell wall in *Ferroplasma* likely increases its reliance on EPS for protection. These EPS, including capsular polysaccharides and lipopolysaccharides, can serve as effective biosorbents for the removal of heavy metals from the environment (Song et al., 2020). The negatively charged functional groups (e.g., carboxyl, sulfate, phosphate) present in lipopolysaccharide and capsular polysaccharide facilitate electrostatic adsorption or complexation with heavy metal ions (Pb^2+^, Cu^2+^, Cd^2+^, etc.) (Ghimire and McCarthy, 2018). Additionally, the formation of biofilms by polysaccharides: lipopolysaccharide and capsular polysaccharide enhances the tolerance of microorganisms to heavy metal stress. However, the structural diversity of polysaccharides: lipopolysaccharide and capsular polysaccharide among different microorganisms results in varying adsorption capacities for heavy metals (Gao et al., 2023). The high molecular weight and negative charge of EPS enable it to adsorb and immobilize heavy metal ions. The presence of numerous metal-binding proteins within MGEs suggests that *Ferroplasma* may secrete these proteins to work in conjunction with EPS, thereby enhancing its capacity to capture heavy metals.

#### 3.4.2 Falcarindiol is a type of QSI

**Figure 7:**
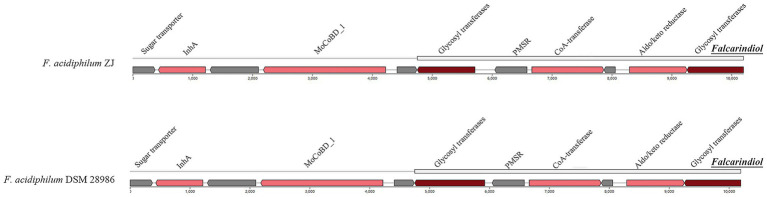
Falcarindiol-related BGC identified from *F. acidiphilum* ZJ and DSM 28986. The core biosynthetic genes, additional biosynthetic genes, and other genes are highlighted in red, pink, and gray, respectively.

As illustrated in Figure 7, the genomes of *F. acidiphilum* ZJ and *F. acidiphilum* DSM 28986 contain an auxiliary BGC responsible for synthesizing falcarindiol, a characteristic acetylenic lipid compound (Jin et al., 2012). Notably, falcarindiol demonstrates QS-modulatory activity, with experimental evidence showing downregulation of QS-associated genes (*rhlA*, *rhlI*, *pqsA*, and *rhlR*) in *Pseudomonas aeruginosa* (Zhao et al., 2021). This suggests falcarindiol may predominantly target the Rhl-QS system and the PQS system in acidophiles. The Rhl-QS system, extensively conserved in *Acidithiobacillus* species, contains a putative LuxI/R homolog pair that facilitates the production of the C_4_-HSL signaling molecule. While the PQS system remains undocumented in acidophiles, genomic analyses reveal widespread distribution of PQS biosynthetic genes *phnA* and *phnB* among AMD-associated acidophiles (Huang et al., 2022).

Multiple sequence alignment of PhnA and PhnB homologs in *Ferroplasma* (Supplementary Figure S3) identified critical conserved residues within their active sites: PhnA contains signature residues G_49_, E_55_, G_58_, I_64_, F_66_, K_68_, L_73_, and P_120_, while PhnB features I_73_, Y_74_, E_118_, G_121_, I_127_, R_129_, K_131_, and L_136_. Notably, the genomic region associated with falcarindiol biosynthesis encodes an “Aldo/keto reductase family oxidoreductase” (Pfam domain), suggesting this enzyme family may play a pivotal role in falcarindiol synthesis, potentially coupled with IS-mediated genetic transfer. Combined with evidence of intrageneric HGT, these findings imply that falcarindiol biosynthesis could exert broad ecological impacts on environmental acidophiles, potentially enhancing the adaptive capacity and ecological competitiveness of *Ferroplasma* archaea through QS modulation.

**Figure 8:**
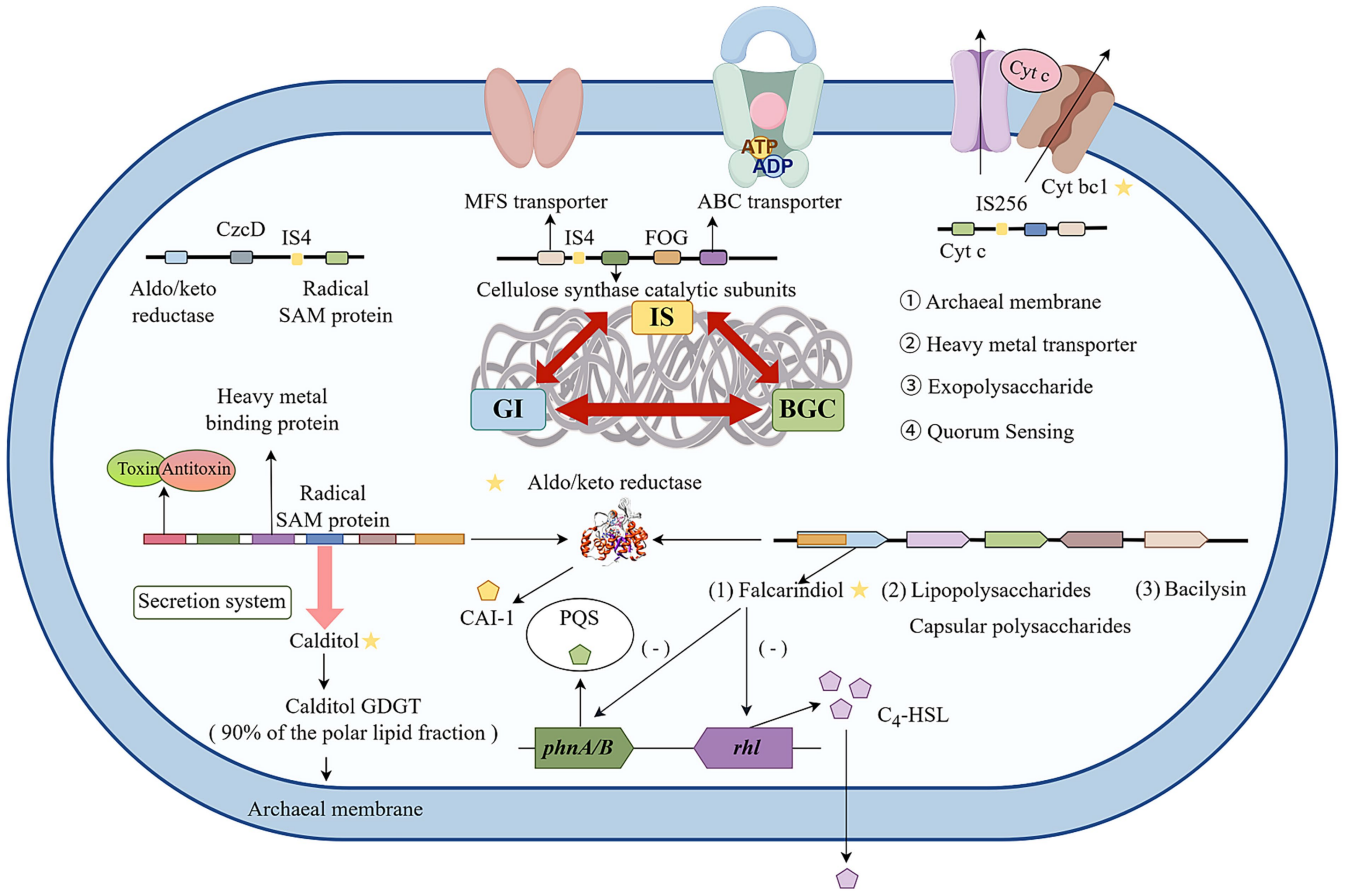
Functional repertoires of the *Ferroplasma*. The MGEs, BGCs, and their possible products were identified in this study. The yellow pentagrams represent archaea-specific genes or substances.

The archaeon *Ferroplasm*a employs specialized MGEs and BGCs as key environmental adaptation mechanisms, as illustrated in Figure 8. Our findings imply that MGEs may mediate HGT, thereby affecting QS regulatory networks, although this link requires further substantiation (e.g., GC content anomalies or phylogenetic incongruence). *Ferroplasma* engages in the redox cycles of metals like iron and sulfur via transporters and invertases, facilitating environmental remediation. Similar to other acidophilic bacteria, its QS system may enhance EPS secretion in response to certain levels of organic carbon or metal ions by regulating EPS synthesis gene transcription (Luo et al., 2024). This mechanism aids biofilm formation, shielding microorganisms from extreme environmental harm, and boosts remediation efficiency through EPS’s heavy metal ion complexation. Thus, EPS synthesis genes are pivotal in *Ferroplasma*’s environmental remediation. Furthermore, QS may optimize microbial metabolic division of labor, addressing resource competition in complex environments by modulating iron oxidation gene expression (Huang et al., 2022). Additionally, its unique membrane lipids, such as calditol-linked glycerol dialkyl glycerol tetraethers, ensure cell stability in highly acidic conditions, supporting sustained remediation efforts. The study revealed substantial genetic diversity and secondary metabolite production capabilities within *Ferroplasma* genomes, highlighting their biotechnological potential for targeted metabolite discovery through systematic genomic exploration.

## 4 Conclusion

The acidophilic archaeon *F. acidiphilum* ZJ was isolated from AMD at the Zijinshan copper mine, China. The present study examined the genomic adaptation strategies of the acidophilic archaeon *F. acidiphilum* ZJ and its closely related strains in the context of extremely polluted environments, such as AMD. The findings highlight the central role of MGEs in driving the adaptive evolution of *Ferroplasma*. These MGEs facilitate the acquisition and integration of key functional genes through HGT, including those encoding heavy metal efflux systems (e.g., CzcD) and the biosynthetic pathway of archaeal ether lipids, which are critical for survival in these harsh, acidic conditions. Our genomic analysis shows that *Ferroplasma* strains possess a diverse array of BGCs, enabling them to produce specialized metabolites, including antibiotics, EPS, and QSI like falcarindiol. Falcarindiol may influence the structure and function of microbial communities in AMD biofilms by disrupting bacterial QS, specifically targeting Rhl and PQS pathways, thus affecting biogeochemical cycles and organic pollutant degradation.

Future research should prioritize the functional validation of key BGCs and the engineering of synthetic consortia. Firstly, it is necessary to deeply analyze the interaction mechanisms of QS interfering molecules, such as falcarindiol, encoded by biosynthetic gene clusters within complex microbial communities. This would elucidate their roles as natural QSI in regulating the synergistic effects of pollutant-degrading microbial consortia, providing a theoretical basis for developing novel bioremediation strategies based on microbial communication intervention. Secondly, by leveraging our understanding of gene horizontal transfer mediated by MGEs, key functional modules driving adaptive evolution should be identified and engineered microbial communities constructed via synthetic biology methods. This would optimize their composite remediation capabilities for heavy metals and organic pollutants in acidic environments. Additionally, research on the molecular mechanisms underlying the extreme tolerance of *Ferroplasma* archaea could be extended to explore other extremophiles resources, offering innovative solutions for the global governance of acidic polluted sites and environmental sustainability.

These results reveal the genomic strategies that enable *Ferroplasma*’s environmental adaptability, emphasizing the combined roles of MGE-mediated gene transfer and specialized metabolic pathways in extreme AMD ecosystems. The identification of QS interference mechanisms and stress-responsive biosynthetic pathways offers new insights into archaeal survival in acidic, metal-rich conditions.

## Data Availability

The datasets presented in this study can be found in online repositories. The names of the repository/repositories and accession number(s) can be found: https://www.ncbi.nlm.nih.gov/, GCA_050311795.1.

## Footnotes

^1^ https://rast.nmpdr.org/
