# Supplementary figures and images for: Roles of mobile genetic elements and biosynthetic gene clusters in environmental adaptation of acidophilic archaeon Ferroplasma to extreme polluted environments
Source: Front Microbiol. 2025 Jul 31;16:1654373. doi: 10.3389/fmicb.2025.1654373 (PMC12350378; doi:10.3389/fmicb.2025.1654373)

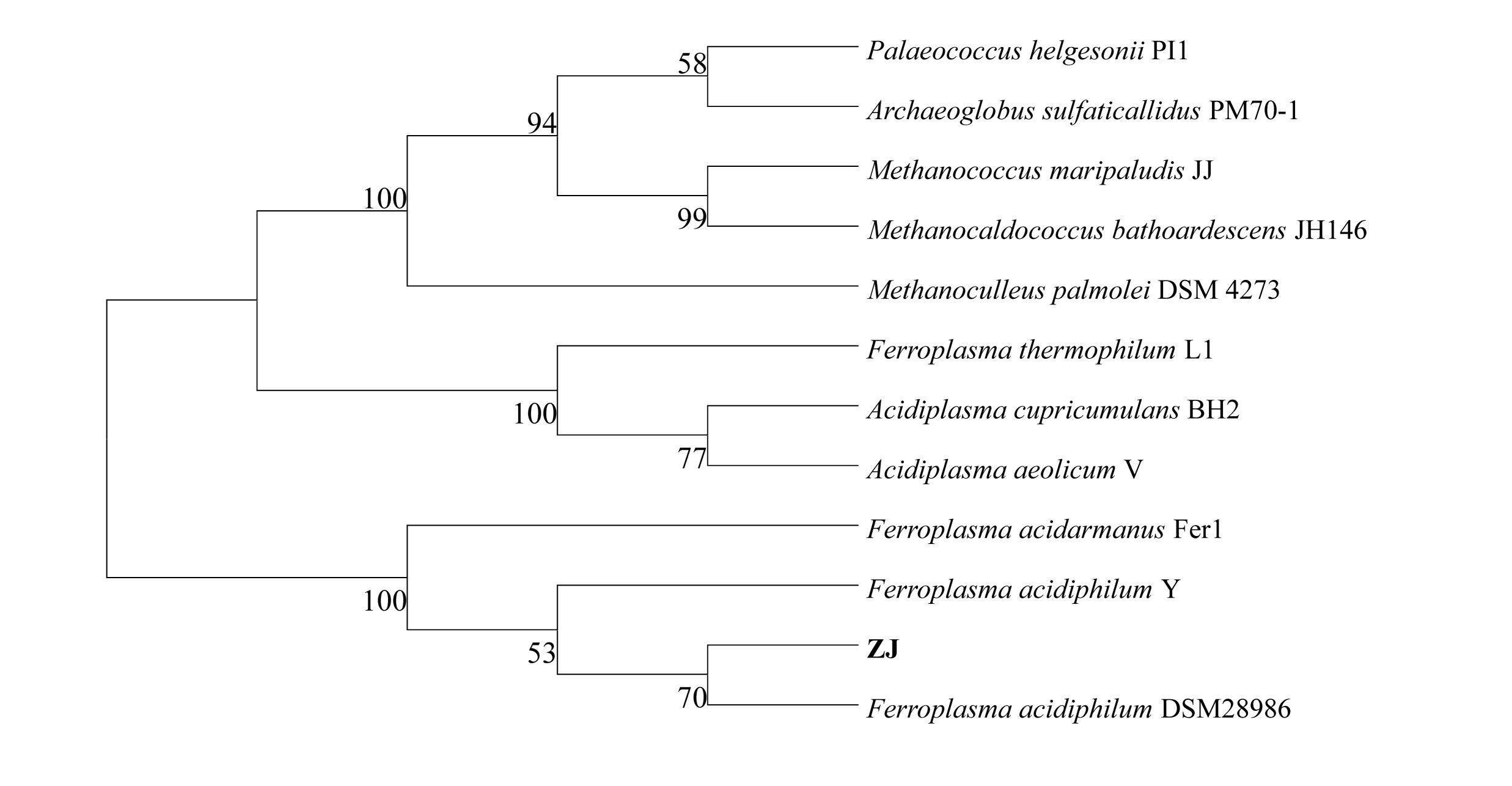

Supplement: Supplementary file 1 [file Image_1.tiff]

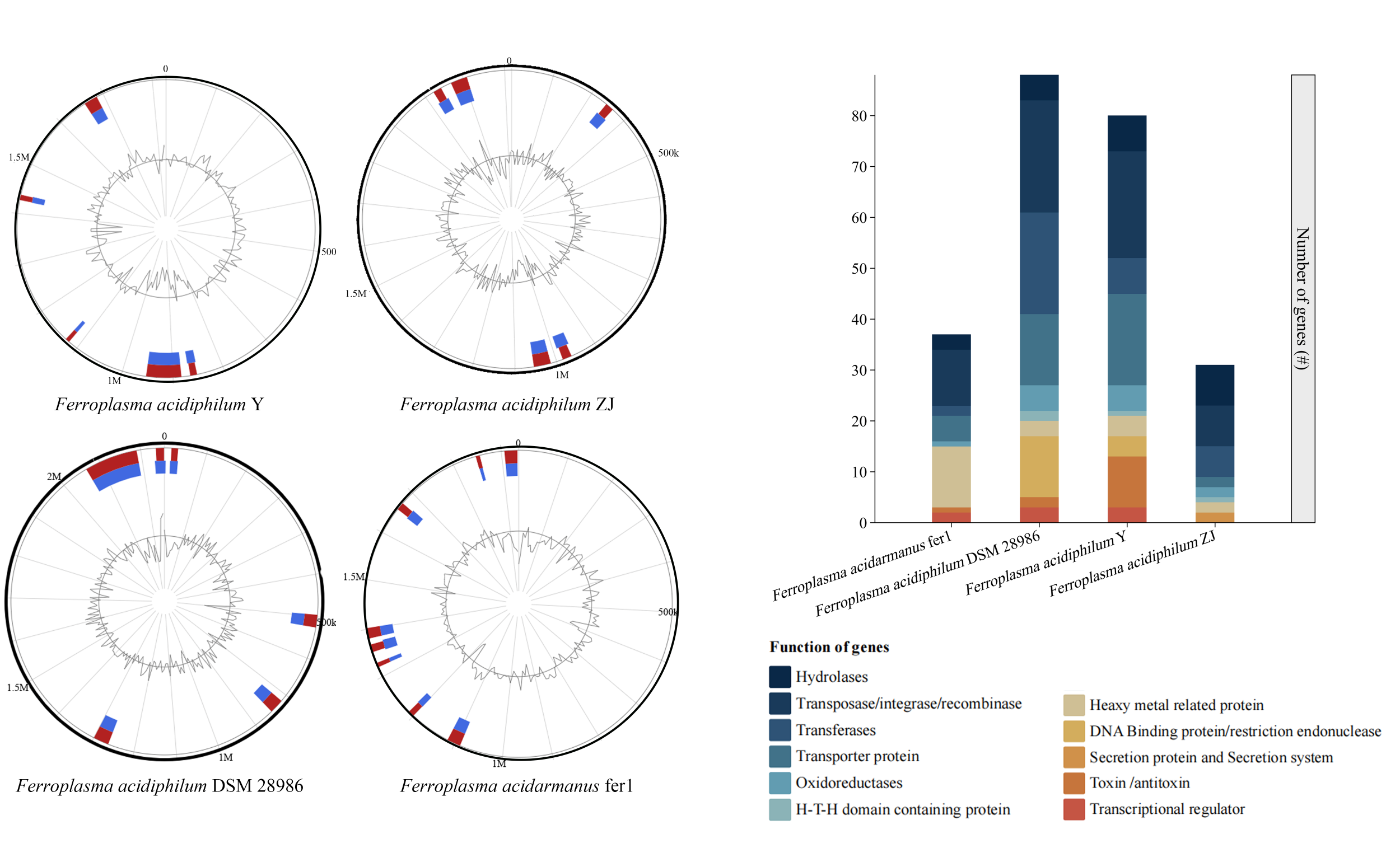

Supplement: Supplementary file 2 [file Image_2.tiff]

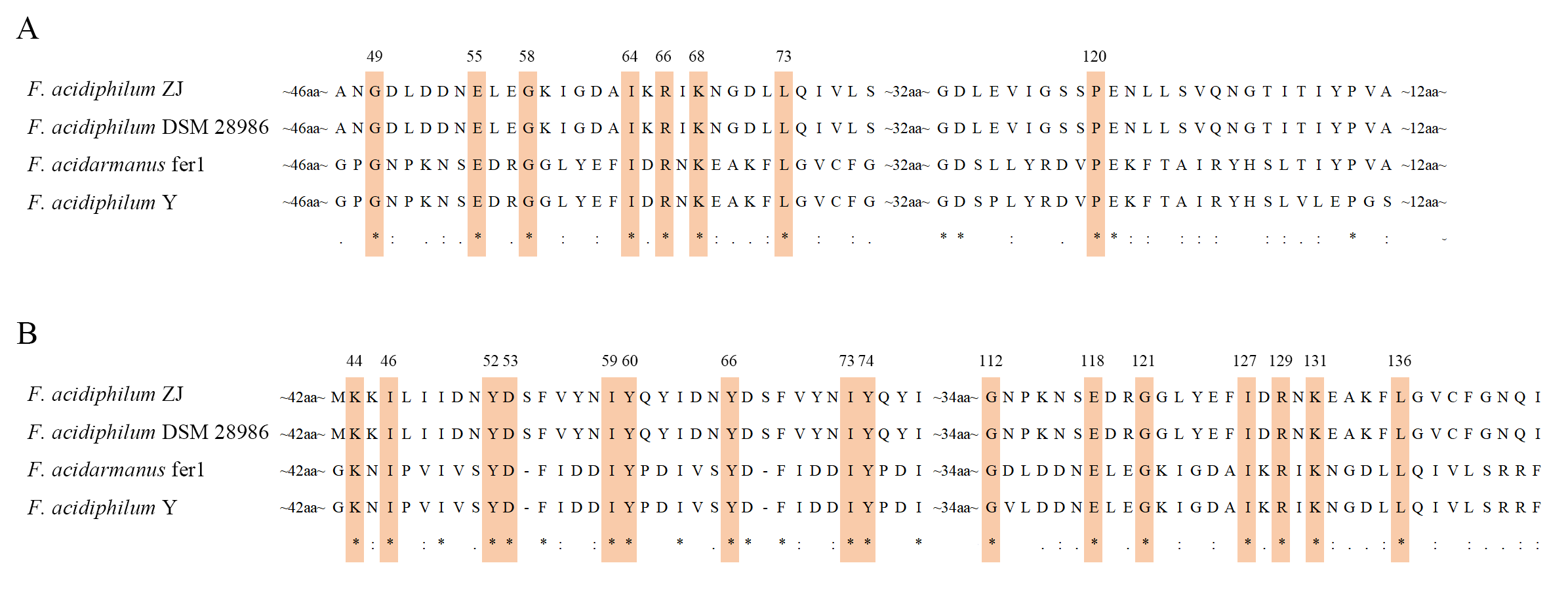

Supplement: Supplementary file 3 [file Image_3.tif]
